# Salivary cardiac-enriched FHL2-interacting protein is associated with higher diastolic-to-systolic-blood pressure ratio, sedentary time and center of pressure displacement in healthy 7-9 years old school-children

**DOI:** 10.3389/fendo.2024.1292653

**Published:** 2024-01-18

**Authors:** Fidanka Vasileva, Raquel Font-Lladó, Gemma Carreras-Badosa, Blanca Roman-Viñas, Aïda Cadellans-Arróniz, Abel López-Bermejo, Anna Prats-Puig

**Affiliations:** ^1^ Pediatric Endocrinology Research Group, Girona Institute for Biomedical Research, Girona, Spain; ^2^ University School of Health and Sport, University of Girona, Girona, Spain; ^3^ Research Group of Culture and Education, Institute of Educational Research, University of Girona, Girona, Spain; ^4^ Department of Physical Activity and Sport Sciences, Blanquerna-Universitat Ramon Llull, Barcelona, Spain; ^5^ Department of Physiotherapy, Faculty of Medicine and Health Sciences, International University of Catalonia, Barcelona, Spain; ^6^ Department of Medical Sciences, University of Girona, Girona, Spain; ^7^ Pediatric Endocrinology, Dr. Josep Trueta Hospital, Girona, Spain; ^8^ Research Group of Clinical Anatomy, Embryology and Neuroscience, Department of Medical Sciences, University of Girona, Girona, Spain

**Keywords:** cardiac-enriched FHL2-interacting protein (CEFIP), cardiovascular markers, children, physical (in)activity, physical fitness, saliva

## Abstract

**Introduction:**

Cardiac-enriched FHL2-interacting protein (CEFIP) is a recently identified protein, first found in the z-disc of striated muscles, and related to cardiovascular diseases. Our objectives are: 1) to quantify CEFIP in saliva in healthy 7-9 years old school-children; and 2) to assess the associations of salivary CEFIP concentration and blood pressure, physical (in)activity and physical fitness in these children.

**Methods:**

A total of 72 children (7.6 ± 0.3 years) were included in the study, recruited in primary schools in Girona (Spain). A sandwich enzyme-linked immunosorbent assay was used (abx506878; Abbexa, United Kingdom) to quantify CEFIP in saliva. Anthropometric evaluation was performed [body mass, height and body mass index (BMI)]. Systolic and diastolic blood pressure were measured by means of an electronic oscillometer and the diastolic-to-systolic blood pressure ratio (D/S BP ratio) was calculated. Physical (in)activity [sedentary time and time spent in physical activity (PA)] were assessed by means of a triaxial Actigraph GT3X accelerometer (Actigraph, Pensacola, FL, USA) that children were instructed to wear for 24h during 7 conssecutive days. Finally, physical fitness (speed and agility, explosive power of legs, handgrip strength, flexibility and balance) were assessed through validated and standardized testing batteries.

**Results:**

CEFIP was easily detected and measured in all saliva samples (mean concentration: 0.6 ± 0.2 pg/ml). Salivary CEFIP was positively associated with D/S BP ratio (r=0.305, p=0.010) and sedentary time (r=0.317, p=0.012), but negatively associated with PA in 7-9 years old school-children (r=-0.350, p=0.002). Furthermore, salivary CEFIP was related to lower level of balance i.e., higher center of pressure (CoP) displacement in these children (r=0.411, p<0.001). The associations of salivary CEFIP with D/S BP ratio (Beta=0.349, p=0.004), sedentary time (Beta=0.354, p=0.009) and CoP displacement (Beta=0.401, p=0.001), were maintained significant after adjustment for potential confounding variables such as age, gender and BMI in linear regression analyses.

**Conclusion:**

CEFIP can be easily assessed in saliva as a promising biomarker associated with cardiovascular health in 7-9 years old school-children. Interestingly, higher salivary CEFIP concentration was related to higher D/S BP ratio, more sedentary time and higher CoP displacement i.e., lower level of balance in these children.

## 1 Introduction

Cardiac-enriched FHL2-interacting protein (CEFIP) is encoded by the *c10orf71* gene on chromosome 10. CEFIP is a recently identified protein found in the z-disc of striated muscles (1, 2). The z-disc is a structural component of both, cardiac and skeletal muscles, placed at the lateral borders of the sarcomere (3). The main function of the z-disc is providing contractility and mechanical stability of striated muscles (4). According to previous evidence which is scarce, CEFIP belongs to a group of highly dynamic z-disc proteins involved in cardiomyocyte and myofibrillar function, mechanosensing and signaling (1, 4). It has been reported that increased CEFIP concentration in cardiac muscle can be observed in various cardiovascular diseases (1). Authors of the previous study described that CEFIP is up-regulated in murine models of heart failure and cardiac hypertrophy, as well as in human ischemic patients, and patients with dilated cardiomyopathy (1).

A recent study reported that overexpression of CEFIP may compromise the proper functioning of the z-disc (1). As previously discussed, the z-disc is involved in producing effective contractions and maintaining stability within the striated muscles (4). Therefore, altered functioning of the z-disc due to CEFIP overexpression may reduce contractility and mechanical stability of striated muscles (1). Reduced contractility and stability of the myocytes in the cardiac muscle may lead to alterations in blood pressure, as well as diastolic dysfunction (5). Diastolic-to-systolic blood pressure (D/S BP) ratio is a relevant indicator of diastolic dysfunction, and is also considered to be a cardiovascular risk marker (6).

In addition, reduced contractility and stability of the myofibrils in the skeletal muscle may diminish physical fitness levels (7). Indeed, it is widely recognized that physical fitness levels are highly dependent on the contractile properties of skeletal muscles (7). Therefore, any alterations within the myofibrils, which are the functional units responsible for skeletal muscle contraction, may significantly impact an individual’s overall physical fitness (3, 4, 7). Physical fitness encompasses a range of skill- and health-related motor abilities including: speed and agility, explosive power of legs, strength, flexibility and balance (8). Speed and agility refer to the ability to produce quick and precise contractions necessary to adapt to quick change of directions, facilitating rapid displacement (9). Explosive power of legs is the ability of the lower body muscles to produce rapid and powerful contractions enabling the generation of maximal force in a very short amount of time (10). Strength represents the capacity of skeletal muscles to generate force during contraction (11). Flexibility is the ability of muscles, tendons, ligaments, and other soft tissues to elongate or stretch, with the aim to increase the range of motion around a joint or series of joints without causing any damage (12). Balance refers to the ability of muscles to maintain postural stability, while the center of pressure (CoP) remains within the base of support, preventing excessive shifts that could lead to instability or falls (13, 14). Aforementioned components of physical fitness are significantly influenced by the individual’s level of physical activity (PA) (8). PA is any bodily movement that increases energy expenditure and is primarily driven by the contraction of skeletal muscles (15). Therefore, alterations in the contractility of myofibrils within skeletal muscles induced by CEFIP overexpression might also affect an individual’s capacity to engage in PA, potentially influencing their overall physical fitness (7).

Hence, understanding the relation between CEFIP, D/S BP ratio, physical fitness and PA is crucial to uncover how alterations at the molecular level can have broader implications on an individual’s ability to perform various activities, impacting their overall health and well-being.

Taking in consideration the previous findings, literature gap and the relevance of this topic, we postulated the following hypotheses and objectives for the present study:

1) Since previous work reported that the protein-coding gene for CEFIP is also expressed in salivary glands (2), we hypothesize that CEFIP can be identified and quantified in saliva in 7-9 years old school-children;
2) Since CEFIP overexpression has been shown to compromise the proper functioning of the z-disc which is involved in providing contractility and stability of the myocytes in the cardiac muscle (1, 4), and altered myocyte contractility may lead to diastolic dysfunction and alterations in the blood pressure (5), we hypothesize that CEFIP may also be related to diastolic dysfunction and blood pressure in 7-9 years old school-children;
3) Furthermore, since any potential alterations in contractility and stability of the myofibrils in the skeletal muscle due to CEFIP overexpression (1, 4) may diminish physical fitness levels (7), and physical fitness is highly dependent on the level of PA (8), we hypothesize that CEFIP may be potentially related to physical (in)activity and physical fitness in 7-9 years old school-children as well.

Therefore, our objectives in healthy 7-9 years old school-children are: 1) To quantify CEFIP concentration in saliva; 2) to explore the association between salivary CEFIP concentration and D/S BP ratio; and finally 3) to explore the associations of salivary CEFIP concentration with physical (in)activity (sedentary time and time spend in PA) and physical fitness (speed and agility, explosive power of legs, handgrip strength, flexibility and balance).

## 2 Methods

### 2.1 Population and ethics

A total of 72 healthy children (7.4 ± 0.3 years) were included in the study, all recruited in schools in Cassà de la Selva and Salt (Girona, Northeastern Spain) meanwhile participating in the ‘‘Physical Education, Children and Health’’ project (details are presented in **Supplementary Figure 1**). Inclusion criteria were: 1) no evidence of chronic or acute illness in the month preceding potential enrollment; and 2) age between 7 and 9 years. We focus on this age range mainly because it serves as a foundational period shaping children’s physical development, as well as establishing habits related to PA (16–20). PA-related habits established during this period tend to be sustained throughout life, thus offering long-term health benefits for these individuals (17, 19). Furthermore, this period witnesses significant strides in motor development including an increase in physical fitness levels (20). Therefore, we believe that this period is vital for fostering healthy growth in children and forming a positive outlook on life. Exclusion criteria were: 1) major congenital abnormalities; 2) chronic illness or chronic use of medication; and 3) musculoskeletal, neurological disorder and/or certain medication therapy that could alter postural stability. The research was approved by the Institutional Review Board of Dr. Josep Trueta Hospital, Girona, Spain. Signed consent was obtained from the parents of all children included in the study. All measurement procedures, evaluations, assessments and sample collection took place on the same day, following enrollment and after obtaining the signed consents for all children included in the study. Prior to any measurement, evaluation or assessment, the children were familiarized with the test protocols. Moreover, they had practice trials before starting the test in order to ensure accurate execution of each test. Sixty percent (%) of the children included in the study were complying with the recommendation for physical activity (≥1 hour/day) (15), while 40% were not.

### 2.2 Biological samples collection

Saliva samples were collected in the morning between 8.00 and 10.00 AM and stored at -20°C following the manufacturer’s protocol. Participants had to discharge 1-4 ml of saliva into the 5 ml polystyrene specimen tube with lid, after natural accumulation in the oral cavity.

### 2.3 Laboratory analysis

To assess salivary CEFIP concentration, a sandwich enzyme-linked immunosorbent assay was carried out by means of ELISA kit (abx506878; Abbexa, United Kingdom). The ELISA kit has the antibody pre-coated onto a 96-well plate. First, the saliva samples were spinned according to the manufacturer’s protocol. Then, standards, saliva samples, and biotin-conjugated reagent were added to the wells and incubated. Next, HRP-conjugated reagent was added, and the whole plate was incubated again. Unbound conjugates were removed using wash buffer at each stage. TMB substrate was used to quantify the HRP enzymatic reaction, and the acidic stop solution was used to stop the reaction. The optical density was measured spectrophotometrically at 450 nm in a microplate reader, from which the concentration of CEFIP was finally calculated in duplicate samples. Intra- and interassay CVs <4%.

### 2.4 Anthropometric evaluation and blood pressure

Anthropometric characteristics were measured in the schools during the morning hours (between 8.00 and 10.00 AM). Body mass was measured wearing light clothes with a calibrated digital scale (Portable TANITA, 240MA, Amsterdam, Netherlands). Height was measured with a wall mounted stadiometer (SECA SE206, Hamburg, Germany). BMI was calculated as body mass in kilograms divided by the square of height in meters. Age- and gender-adjusted standard deviation scores (SDS) for body mass, height and BMI were calculated using regional normative data (21).

Blood pressure was measured in a supine position on the right arm by means of an electronic oscillometer (Dinamap ProCare 100, GE Healthcare) with cuff size appropriate for the arm circumference. The average of three measurements was considered, and finally, D/S BP ratio was calculated.

### 2.5 Physical (in)activity assessment

Triaxial Actigraph GT3X accelerometers (Actigraph, Pensacola, FL, USA) were used to assess sedentary time and time spent in PA (22), mainly because previous research, including recent systematic reviews evaluating the accuracy of measurement tools for physical (in)activity assessment, reported that Triaxial Actigraph devices (in particular GT3X) are accurate, valid and reliable tools for measuring sedentary time and PA in children (23–26).

Initially, the parents received the accelerometers along with comprehensive instructions and wear-time calendars for their children. The children were instructed to wear the accelerometer on an elastic belt at the hip for seven consecutive days. They were allowed to remove it during shower time or swimming activities. Parents were responsible for monitoring their children’s accelerometer wear-time. They were instructed to indicate the time their children went to bed, the time they woke up at the morning, the time they took the accelerometer off, and the time they put the accelerometer on, within the wear-time calendar. Accelerometers were programmed to start collecting data at midnight after they were delivered to the children. The Actilife software (ActiGraph LLC, Pensacola, Florida, USA) was used to initialize, download, and analyze data. Accelerometers were set to a sampling rate of 100Hz, an epoch length of 15 seconds, and a normal filter. After the assessment period, the accelerometers and wear time calendars were returned back to the researchers for data download and analysis. Collected data were reintegrated to 60-second epochs to perform the analysis. Data were screened applying standard accelerometer evaluation methods (27). Non-wear time was identified as 20 consecutive minutes of zero counts. Daily wear time was calculated by subtracting non-wear time from 24 hours. Note that sleep periods and non-wear time were excluded from further analysis. Only children with registers that contained at least four days (with a minimum of one weekend day) of 10 hours valid wear-time were considered for further analysis at the present study. Finally, sedentary time and PA were defined according to the following criteria (28): 1) sedentary time (0 - 100 counts/minute); 2) PA (≥ 101 counts/minute).

### 2.6 Physical fitness assessment

Shuttle run 10 x 5 m test was used to assess speed and agility (29). The shuttle run 10 x 5 m test is a valid and widely used dynamic assessment, involving repeated back-and-forth shuttles over a 5-m distance, summing up to cover a total of 50 m (29). To conduct this test in accurate and safe testing conditions, essential equipment such as a stopwatch, measuring tape, marker cones, and a flat non-slip surface were required. Initially, the designated test area was marked by the researchers with marker cones and lines which were set 5 m apart. Prior to the test, children were given comprehensive instructions on the test protocol by the researcher to ensure accurate execution. To perform the test, the children were instructed to stand behind the starting line, and upon the researcher’s signal they had to run to the second line, and turn and return 10 times continuously without any pause in between. During the test, the researcher had to ensure that the child was crossing the marked line at each shuttle turnaround point with both feet entirely. In case the child did not cross the marked line with both feet entirely at the shuttle turnaround point, they were interrupted immediately, and then the test was repeated to ensure accuracy. The total time (in s) taken to complete the entire 50-m course was recorded with a stopwatch by the researcher to evaluate children’s speed and agility.

Explosive power of legs was assessed with a standing long jump test (29). The standing long jump test is a commonly used test, i.e., a gold standard to evaluate the explosive power of legs (29). To conduct this test accurately, a measuring tape is needed to measure the distance jumped, along with a non-slip surface and a soft landing area, i.e., a mat. Initially, the researchers marked the takeoff line, as well as prepared the soft landing area by placing a mat on the non-slip surface to ensure safety conditions during the test. Before the testing procedure started, children were given comprehensive instructions by the researcher on the test protocol. Also, they had practice trials to ensure proper technique and execution of the test. To perform the test, children were standing with feet slightly apart behind the marked takeoff line on the surface. They were instructed to use a two-foot takeoff technique while initiating the movement from flexed knees position to full knees extension, accompanied by an arm-swing to generate the maximum forward propulsion, and finally land on their feet. The goal was to jump as far as possible while landing on both feet without any backward movement or falling. In case the child attempted to use an additional step before the takeoff, did any backward movement while landing or simply fell, the test was repeated until accurate execution. Scoring entailed measuring the distance (in cm) from the takeoff line to the point where the back of the heels made contact with the landing area. The test was performed twice, and the better result (longer jump) was considered for analysis.

Handgrip strength was assessed by means of an analog hand dynamometer (TKK 5001, Grip-A, Takei, Tokyo, Japan). The handgrip strength test aims to measure the maximum isometric strength of the hand and forearm muscles (in kg), and it is a commonly used indicator for overall strength (29). Before conducting the test, children were given comprehensive instructions by the researcher on the test protocol. Also, the dynamometer grip span was adjusted to 5 cm in order to accommodate children’s hand size and allow the child to comfortably grasp the dynamometer with their fingers wrapped around the handle and the base placed on the heel of their palm (29). To perform the test, children were instructed to hold the dynamometer in their hand while the upper arm and the forearm were forming an angle of approximately 90°, and their elbow was kept beside the body (29). The goal was to squeeze the dynamometer with maximum isometric force which had to be maintained for 5 s (29). While exerting maximum pressure on the dynamometer, children had to refrain from doing any additional body movements or adjustments. During the test, the researcher was responsible to ensure that the children were performing the test accurately, i.e., that the dynamometer base was placed on the heel of the palm, while the handle on the middle of the four fingers. Also, the researcher encouraged the children to exert their maximum effort during the test. In case the child did any adjustment or unnecessary movement during the test, the test was repeated until accurate execution. The test was performed twice, and the better result (higher value) was considered for analysis.

Lower back and hamstrings flexibility was assessed by means of a sit-and-reach test (29). The sit-and-reach test is widely used to assess flexibility of lower back and hamstring muscles, and is also considered a general indicator of flexibility (29). To conduct the sit-and-reach test, a standard sit-and-reach box and a mat were necessary. The researcher began the setup by laying out a mat on the floor and positioning the sit-and-reach box in contact with the wall to ensure stability and proper alignment for conducting the test. Before starting the test, the researcher explained the testing procedure to the children. To perform the test, children were instructed to sit on the floor without shoes, with legs stretched out straight while both knees were locked, and their feet placed against the front side of the box. Then, they were told to situate one hand on the top of the other with palms facing downwards, and to gradually extend both arms forward along the measuring scale on the box. This action involved leaning forward as much as possible. After each child performed a practice trial to assure accuracy of the execution, they were allowed to start the test. The goal of the test was to extend the arms forward without bending the knees, and reach as far as possible on the measuring scale. The children had to hold the final position for at least 2 s while the distance was recorded (in cm). During the test, the researcher had the responsibility to assure that the children were performing the test accurately, i.e., without bending their knees, with the hands remaining at the same level (not one reaching further forward than the other), and without any jerky movements. The test was performed twice, and the better result (longer reach) was considered for analysis.

Balance was assessed by measuring the CoP displacement (in mm) with the closed eyes bipedal test performed on a Wii balance board platform according to the protocol designed for children (13, 14). The closed eyes bipedal test challenges the proprioception by requiring children to maintain their equilibrium without visual input (13). More precisely, when closing their eyes, children rely on their body’s sensory systems to sustain postural stability excluding visual cues. Additionally, the Wii balance board platform comprises a pressure-sensitive surface that detects weight shifts and CoP displacement, thus being a valid and reliable tool, commonly used for balance assessment in research and clinical settings (14). Thus, in order to conduct this assessment with accuracy at the present study, the following special equipment was required: Wii balance board platform, laptop, MatLab software (WiiLab toolbox) and a dark eye-cover. Initially, the researcher prepared the assessment area by placing and connecting the necessary equipment. The Wii balance board platform was connected to a laptop via Bluetooth to be ready for the measurement. Before starting the test, the researcher explained the test protocol to the children. To perform the test, children were instructed to stand in a bipedal position on the platform, ensuring that the distance between their ankle joint centers was equivalent to the distance between their right and left anterior superior iliac spines. Both legs had to be fully extended with the heels aligned and the feet parallel and facing forward. Arms had to be kept relaxed by the sides. After the initial adjustments were completed (5-10 s) and the eye-cover was placed, the assessment was initiated by the researcher who was also responsible to detect any possible irregularity during the assessment (e.g., eye-cover drop, open eyes etc.). The goal was to maintain the standing still position on the board platform with closed eyes for 30 s. The MatLab software (WiiLab toolbox) was used to record the CoP displacement in side-to-side and front-to-back directions registered in mm. In case of irregularity, the assessment was interrupted immediately and the child repeated the test to assure accuracy. The test was performed twice, and the better result (lower CoP displacement value) was considered for analysis.

### 2.7 Statistical analysis

A GRANMO 7.12 program was used in order to identify the sample size for protein concentration assessment. Accepting an alpha risk of 0.05 and a beta risk of 0.2, a minimum of 60 participants were necessary (30).

Data were analyzed with the statistical package SPSS version 22.0 (SPSS Inc, Chicago, IL, United States). The normality of the data distribution was tested by the Kolmogorov-Smirnov test. Data were normally distributed and no outliers were identified. Thus, to explore the associations of CEFIP with D/S BP ratio, physical (in)activity and physical fitness, parametric measures such as bivariate Pearson correlations were applied. Pearson correlation values were interpreted as: 1) low association: r = |0.10 - 0.30|; 2) moderate association: r = |0.31 - 0.50|; 3) high association: r = |0.51 – 0.70|; 4) very high association: r = |0.71 – 0.90|; and 5) almost perfect association: r = |0.91 – 1.00| (31). Statistical significance was set at p<0.05. To correct the identified associations for potential confounding variables such as age, gender and BMI, multiple linear regression analyses were applied using the enter method. Multiple testing Bonferroni correction was also applied to obtain critical level for statistical significance.

## 3 Results

Descriptive characteristics of the studied children are shown in **Table 1**. From the results presented, it can be observed that CEFIP was successfully detected and quantified in all saliva samples (mean concentration 0.6 ± 0.2 pg/ml).

**Table 1:** Descriptive characteristics of the studied population (N=72).

Demographic and anthropometric characteristics	
age (years)	7.57 ± 0.33
gender (m/f)	24/48
body mass (kg)	27.1 ± 4.9
body mass SDS	-0.19 (-0.72 – 0.20)
height (cm)	127 ± 5
height SDS	0.12 (-0.52 – 0.75)
BMI (kg/m²)	16.6 ± 1.9
BMI SDS	-0.23 (-0.82 – 0.21)
Cardiovascular risk markers	
CEFIP (pg/ml)	0.6 ± 0.2
systolic BP (mm/Hg)	103.5 ± 9.8
diastolic BP (mm/Hg)	61.7 ± 8.3
D/S BP (mm/Hg) ratio	0.59 ± 0.07
Physical (in)activity	
sedentary time (hours/day)	6.5 ± 1.7
PA (hours/day)	1.1 ± 0.3
Physical fitness	
shuttle run 10 x 5 m (s)*	25.32 ± 2.51
standing long jump (cm)	93.6 ± 17.6
hand dynamometry (kg)	11.4 ± 2.7
sit-and-reach test (cm)	26.0 ± 8.1
CoP displacement (mm)*	174.31 ± 51.53

Data for Gaussian variables is presented as mean ± standard deviation. Data for non-Gaussian variables is presented as median and interquartile range. BMI, body mass index; BP, blood pressure; CEFIP, cardiac-enriched FHL2-interacting protein; CoP, center of pressure; D/S, diastolic to systolic; PA, physical activity; SDS, standard deviation score; *: variable with an opposite metric orientation.

**Figure 1:**
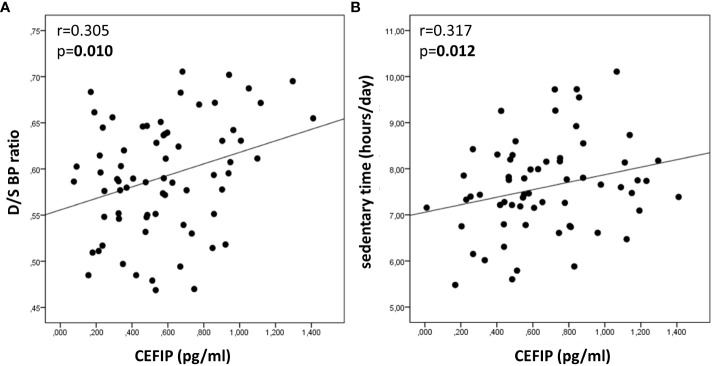
Bivariate associations of salivary CEFIP concentration with D/S BP ratio and sedentary time in healthy 7-9 years old school-children. **(A)** Scatterplot representing the association between salivary CEFIP concentration and D/S BP ratio. **(B)** Scatterplot representing the association between salivary CEFIP concentration and sedentary time. CEFIP: cardiac-enriched FHL2-interacting protein; D/S BP ratio: diastolic-to-systolic blood pressure ratio.

A moderate positive association (r= 0.305, p=0.010) was identified between CEFIP concentration and D/S BP ratio in healthy 7-9 years old school-children (**Figure 1A**). In addition, CEFIP concentration showed a moderate positive association with sedentary time (r= 0.317, p=0.012; **Figure 1B**).

Bivariate associations of CEFIP concentration with PA and physical fitness are presented in **Table 2**. Based on **Table 2**, CEFIP concentration showed a moderate negative association with PA (r= -0.350, p=0.002). Even though no associations were identified between CEFIP concentration and speed and agility, explosive power of legs, handgrip strength and flexibility (**Table 2**), higher CEFIP concentration was related to higher CoP displacement in these children (r= 0.411, p<0.001; **Table 2**).

**Table 2:** Bivariate associations of salivary CEFIP concentration with PA and physical fitness in healthy 7-9 years old school-children (N=72).

	CEFIP (pg/ml)	
	r	p-value
PA (hours/day)	-0.350	**0.002†**
shuttle run 10 x 5 m (s)*	0.099	0.585
standing long jump (cm)	0.056	0.760
hand dynamometry (kg)	0.068	0.580
sit-and-reach test (cm)	-0.186	0.118
CoP displacement (mm)*	0.411	**<0.001†**

Significance level is set at p<0.05 and significant values are marked in bold. Significance level after multiple testing Bonferroni correction is set at p<0.008 and marked with † within the table. CEFIP, cardiac-enriched FHL2-interacting protein; CoP, center of pressure; PA, physical activity; *: variable with an opposite metric orientation.

Multiple linear regression analyses to correct the previously identified associations for potential confounding variables such as age, gender and BMI are presented in **Table 3**. Independent associations between CEFIP concentration and D/S BP ratio (Beta= 0.349, p=0.004, adjusted R^2 =^ 0.119), sedentary time (Beta= 0.354, p=0.009, adjusted R^2 =^ 0.101) and CoP displacement (Beta= 0.401, p=0.001, adjusted R^2 =^ 0.108) were observed in the studied children.

**Table 3:** Linear regression analyses correcting the associations for confounding variables: age, gender and BMI (N=72).

CEFIP (pg/ml)			
	Beta	p-value	Adjusted R^2^
D/S BP (mm/Hg) ratio	0.349	**0.004†**	0.119
sedentary time (hours/day)	0.354	**0.009†**	0.101
PA (hours/day)	-0.124	0.319	-0.009
CoP displacement (mm)*	0.401	**0.001†**	0.108

Corrected for age, gender and BMI. Significance level is set at p<0.05 and significant values are marked in bold. Significance level after multiple testing Bonferroni correction is set at p<0.013 and marked with † within the table. BMI, body mass index; CEFIP, cardiac-enriched FHL2-interacting protein; CoP, center of pressure; D/S BP, diastolic to systolic blood pressure ratio; PA, physical activity; *: variable with an opposite metric orientation.

## 4 Discussion

Main findings of the present study indicate that CEFIP is conveniently quantified in saliva samples from healthy 7-9 years old school-children. In these children, higher salivary CEFIP concentration was related to higher D/S BP ratio, more sedentary time, and higher CoP displacement i.e., lower level of balance.

This is the first study to show that the novel CEFIP protein can be detected and quantified in saliva. The present outcome allows us to accept the first hypothesis of this study. In support to our findings, a previous study reported that the protein-coding gene for CEFIP is expressed in salivary glands (2). In the past decades, the most used diagnostic fluids in laboratory setting were blood and urine (32). However, recent studies that compared the concentration of proteins in saliva and plasma have observed that same components are found in similar concentration in both fluids (33). Thus, nowadays, saliva is becoming an acceptable diagnostic bio-specimen, mainly due to the advancements in protein analytical techniques (34). Saliva collection does not require specially trained personnel for sampling procedure, it is easy, non-invasive and more practical (32). The use of saliva as a diagnostic fluid may facilitate early disease detection and alterations in health, and improve the clinical management (32). In this line, previous studies demonstrated the use of salivary markers in the early detection of a cardiovascular disease (35–38). For instance, salivary cytokines such as IL-6 and TNF-α were suggested as potential markers for atherosclerosis due to their significant increase in atherosclerosis patients (35). In addition, salivary C-reactive protein concentration in combination with an electrocardiogram predicted acute myocardial infarction, reinforcing the idea that salivary markers are effective for an early detection of potential alterations in the cardiovascular health (38).

In the present study, higher salivary CEFIP concentration was related to higher D/S BP ratio in healthy 7-9 years old school-children. This finding confirms the second hypothesis of this study. D/S BP ratio is widely recognized as a relevant cardiovascular disease risk marker (6). Even though the association between salivary CEFIP concentration and D/S BP ratio was explored for the first time in the present work, a previous study has identified a relation between the novel z-disc derived protein CEFIP and cardiac hypertrophy (1). Furthermore, another study showed that cardiac hypertrophy is preceded by diastolic dysfunction (39). Indeed, the z-disc protein CEFIP has been shown to induce cardiomyocyte hypertrophy in mouse models (1). Authors of the previous work suggested that CEFIP may have a role in cardiomyocyte hypertrophy via modulation of the calcineurin-dependent signaling (1). In addition, they also reported that in humans, CEFIP was significantly higher in cardiac tissue from patients with ischemic or dilated cardiomyopathy compared to controls (1). According to previous evidence, CEFIP may act as a mediator in cardiovascular disease, probably stimulated by the biomechanical stress which may potentially induce maladaptive hypertrophic response in the cardiomyocytes (1, 39). Finally, the positive association between salivary CEFIP concentration and D/S BP ratio in healthy 7-9 years old school-children identified in the present study, reinforces the previous findings that reported the role of the novel CEFIP protein in cardiovascular disease. Moreover, our findings may indicate that salivary CEFIP concentration may be potentially considered as a cardiovascular disease risk marker for early detection of possible alterations in cardiovascular health. However, due to the cross-sectional nature of the present study, further longitudinal studies are necessary to clarify the relation between CEFIP and cardiovascular disease, as well as to examine the utility of salivary CEFIP concentration as a non-invasive cardiovascular disease risk marker.

In addition, higher salivary CEFIP concentration was related to more sedentary time in healthy 7-9 years old school-children. There is no previous evidence exploring the association between CEFIP and sedentary time. However, higher CEFIP concentration was previously related to cardiac hypertrophy (1). In addition, a previous study indicated that cardiac hypertrophy was related to more sedentary time (40). Based on previous findings, we assume that the association between sedentary time and higher salivary CEFIP concentration, may be potentially explained by the role of CEFIP in signaling, myofibrillar remodeling and cardiac geometry (1, 41, 42). Even though CEFIP was first identified as a structural component of the z-disc in striated muscles (1), experiments designed to determine the kinetics of diffusion of the protein have revealed extraordinary dynamics and mobility of CEFIP compared to other proteins in mammalian and avian muscle cells (41, 43–46). According to what has been previously reported and taking in consideration that we were able to detect CEFIP in saliva in the present study, it can be herein suggested that CEFIP may have a significant role in tissue signaling, rather than just being a structural protein within the myofibril (1). Further studies are necessary to elucidate the specific role of CEFIP in saliva.

Present findings also indicate that salivary CEFIP concentration is related to higher CoP displacement, i.e., lower level of balance in healthy 7-9 years old school-children. No previous studies have explored the association between CEFIP and balance. We believe that the association identified in the present study may be related to the negative alteration in the primary function of the z-disc which is providing mechanical stability within the muscle (4), potentially induced by increased CEFIP concentration (1). It is clear that in the present study we cannot assess CEFIP concentration in skeletal muscle tissue in children due to ethical reasons. However, previous studies reported that the z-disc derived protein CEFIP is highly dynamic protein which may be participating in important signaling among tissues (4, 41, 43–46). Thus, further studies will be necessary to elucidate the molecular pathways that are explaining CEFIP signaling and tissues-crosstalk. What is already known is that the z-disc is a structural constituent of the sarcomere which is important for the cross-linking of thin filaments and the effective transmission of force generated within the muscle (4). Any perturbations of the functional interaction of the myofibrillar components (4), possibly induced by alterations in the concentration of the z-disc proteins (1), may lead to disruption and malfunction of the contractile apparatus, as well as negatively altered mechanical stability within the muscle (47). Therefore, we hypothesize that higher CEFIP concentration in the present study may have probably induced negative alterations in the mechanical stability within the muscles as reported in previous studies (1, 47), potentially leading to higher CoP displacement and lower level of balance in these children. However, as highlighted previously, further studies should clarify the relation between CEFIP and CoP displacement and reinforce the findings of the present study.

Finally, the current findings allow us to partially accept the third hypothesis because we observed an association between salivary CEFIP concentration and sedentary time, but we did not observe associations with all components of physical fitness. More precisely, salivary CEFIP concentration was related only to balance, and was not related to speed and agility, explosive power of legs, handgrip strength and flexibility in 7-9 years old school-children.

## 5 Conclusion

The recently identified protein CEFIP which was first found in the z-disc of striated muscles can be certainly detected and conveniently quantified in saliva. Interestingly, higher D/S BP ratio and more sedentary time were related to higher salivary CEFIP concentration, and higher salivary CEFIP concentration was related to higher CoP displacement i.e., lower level of balance in 7-9 years old school-children.

Present findings are indicating that salivary CEFIP may be potentially considered as a cardiovascular disease risk marker for early detection of possible alterations in cardiovascular health in children. Moreover, it seems that more sedentary time is related to an increase in salivary CEFIP concentration. Therefore, we recommend a reduction of the sedentary time and an increase in PA among children with the aim to prevent any possible cardiovascular alterations and maintain optimal health.

### 5.1 Practical applications

It is noteworthy that in the current study we successfully measured CEFIP concentration in saliva. This achievement holds particular significance especially for diagnostic purposes in children, mainly because saliva collection is a non-invasive procedure. Hence, saliva as a non-invasive diagnostic fluid could offer valuable insights into children’s cardiovascular health, and an early detection of any possible alterations. This may potentially lead to efficient and proactive management of cardiovascular issues in pediatric populations.

Current findings may also encourage practicing more PA and reducing the sedentary time in order to maintain the cardiovascular health in children. This insight could potentially motivate future modifications in Physical Education Curricula, advocating for a higher frequency of classes per week, with the aim to promote health and contribute to disease prevention in school-children.

### 5.2 Limitations and future research

Even though the cross-sectional design of the present study may be a potential limitation, the study design corresponds to a proof-of-concept study and may serve as a starting point for future research.

The present findings highlight the necessity for developing longitudinal studies that will examine the utility of salivary CEFIP concentration as a cardiovascular disease risk marker. Long-term investigations that follow individuals over extended periods could offer valuable insights into the consistency and predictive capacity of salivary CEFIP concentration in relation to the development or progression of cardiovascular diseases over time. Understanding the predictive potential of CEFIP in saliva could have significant implications for early detection, prevention, and management strategies related to cardiovascular health.

Experimental studies and basic research are also needed to elucidate the specific role of CEFIP in saliva and the molecular pathways involved in tissue cross-talk and signaling. These studies could provide crucial insights into its function and potential implications in health and disease.

Additionally, further experimental studies and basic research should aim to clarify the role that CEFIP may have in negative alteration of the mechanical stability within the muscles. It is crucial to understand whether CEFIP can impact the performance of the central nervous system, or impact the fine motor skills of an individual. Exploring the specific mechanisms through which CEFIP might contribute to negative alterations in the mechanical stability within muscles could provide novel insights into disease mechanisms and the development of potential therapeutic interventions aimed at preserving muscle function and stability.

Besides studies in saliva, another interesting point for future research may be quantifying serum CEFIP concentration in humans, and exploring its relation with cardiac muscle and vasculature. Understanding how CEFIP concentration in the bloodstream correlates with cardiac muscle function and vascular health, holds promise for uncovering additional markers or indicators related to cardiovascular health and disease. This research path could offer valuable insights into the role of CEFIP in cardiovascular physiology and its potential implications for cardiac and vascular conditions. Consequently, this may possibly pave the way for novel diagnostic and therapeutic avenues in cardiac and vascular medicine.

Additional research through animal studies should encompass quantifying CEFIP concentration specifically within skeletal muscle. These investigations should delve deeper into understanding the role of CEFIP in relation to mechanical stability, contractility, and overall muscle function, providing crucial knowledge regarding its functional significance in muscle health and performance. By quantifying CEFIP in skeletal muscle and investigating its role in mechanical stability and muscle function through animal studies, researchers could gain a clearer understanding of the potential impact of CEFIP on muscular physiology. These findings may have implications for elucidating its role in muscle-related conditions and potentially guide the development of therapeutic approaches aimed at preserving or enhancing muscle health.

Further interventional studies should compare the effects of various types of physical exercise with different intensities, volumes, and frequencies on both salivary and serum CEFIP concentrations. These studies could shed light on the potential influence of exercise on CEFIP regulation within the body, and elucidate how exercise intensity, duration, and frequency affect CEFIP concentration, offering valuable information about the physiological responses to varying exercise regimens. Such studies could contribute significantly to optimizing exercise prescriptions tailored to modulate CEFIP concentration, potentially influencing overall health and fitness outcomes.

Finally, it would be beneficial to explore gender-specific differences in CEFIP concentration. Future research employing larger sample size should focus on comparing CEFIP concentration in males and females, as well as exploring associations between CEFIP and various health and physiological parameters in males and females independently, to better understand potential gender-specific implications. Indeed, investigating how CEFIP relates to specific health outcomes in males and females could provide valuable insights into its role in different biological functions.

## Data availability statement

The original contributions presented in the study are included in the article/**Supplementary Material**. Further inquiries can be directed to the corresponding author.

## Ethics statement

The studies involving humans were approved by Institutional Review Board of Dr. Josep Trueta Hospital, Girona, Spain. The studies were conducted in accordance with the local legislation and institutional requirements. Written informed consent for participation in this study was provided by the participants’ legal guardians/next of kin.

## Author contributions

FV: Conceptualization, Formal analysis, Investigation, Methodology, Software, Visualization, Writing – original draft. RF-L: Conceptualization, Data curation, Formal analysis, Funding acquisition, Investigation, Methodology, Project administration, Software, Supervision, Writing – review & editing. GC-B: Data curation, Formal analysis, Investigation, Methodology, Software, Writing – review & editing. BR-V: Data curation, Formal analysis, Methodology, Software, Writing – review & editing. AC-A: Data curation, Formal analysis, Methodology, Software, Writing – review & editing. AL-B: Formal Analysis, Methodology, Supervision, Visualization, Writing – review & editing. AP-P: Conceptualization, Data curation, Formal Analysis, Funding acquisition, Investigation, Methodology, Project administration, Software, Supervision, Visualization, Writing – review & editing.

## Supplementary material

Supplementary Figure 1 Flowchart of enrollment methodology. PEHC: Physical Education, Health and children.
