# Supplementary figures and images for: Salivary cardiac-enriched FHL2-interacting protein is associated with higher diastolic-to-systolic-blood pressure ratio, sedentary time and center of pressure displacement in healthy 7-9 years old school-children
Source: Front Endocrinol (Lausanne). 2024 Jan 18;15:1292653. doi: 10.3389/fendo.2024.1292653 (PMC10830845; doi:10.3389/fendo.2024.1292653)

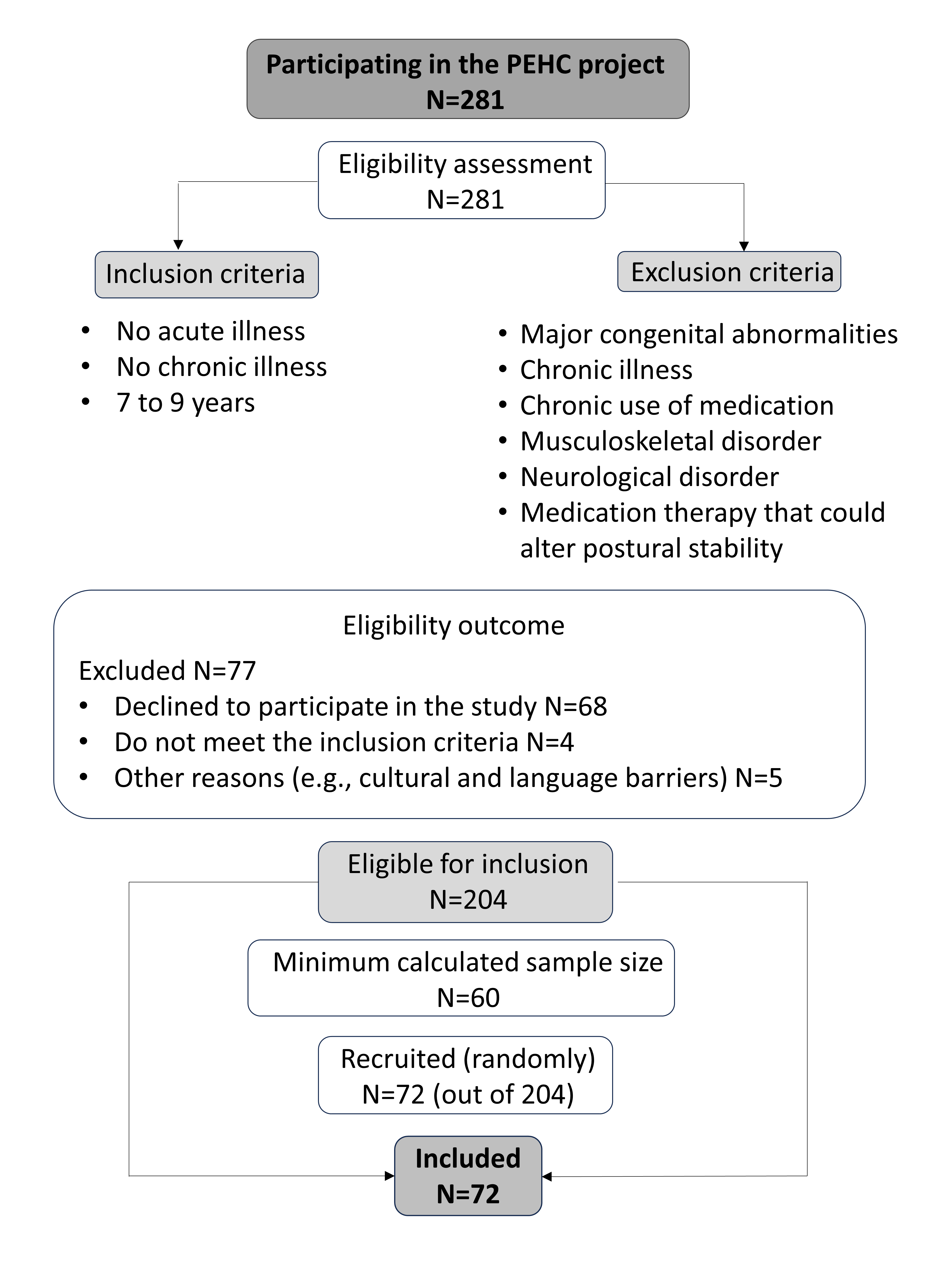

Supplement: Supplementary Figure 1 — Flowchart of enrollment methodology. PEHC: Physical Education, Health and children. [file Image_1.tif]
